# Effect of systematic nursing intervention on rehabilitation after colorectal polyps of endoscopic removal in children: A protocol for systematic review and meta-analysis

**DOI:** 10.1097/MD.0000000000025109

**Published:** 2021-03-26

**Authors:** Zhenyin Dong, Guizhen Li, Meigui Wang, Tingting Wang, Chengpeng Jiang

**Affiliations:** a Department of Pediatric Gastroenterology, Gansu Provincial Maternity and Child-Care Hospital; b Department of Information System, Gansu Provincial Hospital of TCM, Lanzhou, Gansu Province, China.

**Keywords:** colorectal polyps in childhood, endoscopic removal, protocol, system nursing, systematic review

## Abstract

**Background:**

Endoscopic removal is the main method for the treatment of colorectal polyps in children. Due to the small age of children, poor coordination, postoperative sensitive postoperative response, it is not good for postoperative recovery. Systematic nursing has an advantage in promoting the postoperative recovery of children with colorectal polyps of endoscopic removal, but it is lack of evidence-based basis. The purpose of this study is to evaluate the effect of systematic nursing intervention on the rehabilitation of children after colorectal polyps of endoscopic removal.

**Methods:**

China National Knowledge Infrastructure, Wanfang, China Science and Technology Journal Database and Chinese Biomedical Literature Database, PubMed, Embase, Web of Science, and the Cochrane Library databases will be searched by computer. A randomized controlled study is searched on the application of systematic nursing intervention of children with colorectal polyps of endoscopic removal from the establishment of the database in February 2021. The language is limited to English and Chinese. The quality of the included study is independently extracted and the literature quality is evaluated by 2 researchers. The RevMan5.3 software is used to conduct meta-analysis of the included literature.

**Results:**

This study will evaluate the effect of systematic nursing intervention on the rehabilitation of children after colorectal polyps of endoscopic removal by the indexes of total effective rate, complication rate, and hospital stays.

**Conclusion:**

This study will provide reliable evidence-based basis for establishing a reasonable and effective nursing intervention for endoscopic removal of colorectal polyps in childhood.

**OSF Registration number:**

DOI 10.17605/OSF.IO/S57UX.

## 1 Introduction

Colorectal polyps are protuberant lesions of colorectal mucosa from mucous layer protuberance to intestinal lumen. It is a common cause of lower gastrointestinal bleeding in children, and its incidence increases with age.^[1]^ The typical clinical symptom is painless rectal bleeding.^[2]^ Studies from the United States, Pakistan, India, and other countries show that the incidence of colorectal polyps in children is 1% to 1.1%,^[3,4]^ and the differences may be related to regional, population characteristics, medical level, and other factors in different countries. The etiology and pathogenesis of colorectal polyps in children are not clear. Most studies believe that the occurrence of colorectal polyps is related to genetic factors.^[5]^ It is generally believed that colorectal polyps in children have no tendency to become cancerous, but there is still a certain possibility of malignant transformation.^[6]^ At present, the treatment of colorectal polyps in children is advocated to be removed as soon as it is first found. Colorectal polyps of endoscopic removal have replaced surgery because of its simplicity, fewer complications and high safety, and have become the main method for the treatment of colorectal polyps.^[7–9]^

Because the children are young and can not cooperate by themselves, and the children are sensitive to the operation and slight discomfort after the operation, and are prone to cry and other bad emotions. This is very disadvantageous to the preoperative preparation, operation, and postoperative recovery. Therefore, it is necessary to provide relevant physical and psychological nursing to the children and their families in the preoperative period to ensure the smooth implementation of the operation.^[10]^ Systematic nursing intervention refers to systematic health education nursing on the basis of routine nursing, including psychology, diet, exercise, family cooperation, follow-up nursing, and so on. Making the patient's physical, mental, and social function reaches the best state comprehensively and systematically.^[11]^

At present, a number of randomized controlled studies have confirmed that systematic nursing intervention can significantly improve the preoperative traumatic stress response of endoscopic polypectomy in children, reduce the incidence of postoperative complications, and improve the effective rate of treatment.^[12–16]^ However, due to the differences in research schemes and nursing contents, there are differences in research results and lack of reliable evidence-based basis, which limits the promotion of this program. Therefore, this study plans to systematically evaluate the effect of systematic nursing intervention on the rehabilitation of children with colorectal polyps after endoscopic removal, in order to get a standardized and reliable conclusion.

## 2 Methods

### 2.1 Protocol register

This protocol of systematic review and meta-analysis has been drafted under the guidance of the preferred reporting items for systematic reviews and meta-analyses protocols.^[17]^ Moreover, it has been registered on open science framework (OSF) (Registration number: DOI 10.17605/OSF.IO/S57UX).

### 2.2 Ethics

Since the program does not require the recruitment of patients and the collection of personal information, it does not require the approval of the Ethics Committee.

### 2.3 Inclusion criteria

The randomized controlled trial study of all children (≤14 years old) with colorectal polyps who undergo endoscopic removal and the comparison of the results of systematic nursing intervention and traditional nursing intervention is in accordance with the criteria for inclusion in this systematic evaluation.

### 2.4 Exclusion criteria

(1) The published literature is abstract, review, and animal research.
(2) Repeatedly published literature for the same research population.
(3) Articles with incomplete or incorrect data and unable to obtain complete data after contacting the author.
(4) Literature with inconsistent intervention methods or no related outcome indicators.

### 2.5 Outcome indicators

Main outcome measures:

(1) total effective rate;
(2) postoperative complications (including bleeding, abdominal pain, intestinal perforation, etc).

Secondary outcome measures: hospital stays; exsufflation time by anus.

### 2.6 Retrieval strategy

The words “colorectal polyp,” “pediatric polyp,” “systematic nursing,” and “comprehensive nursing” are searched in China National Knowledge Infrastructure, Wanfang, China Science and Technology Journal Database, and Chinese Biomedical Literature Database, while “system nursing,” “nursing intervention,” “colorectal polyps,” “colonic polyps,” and “endoscopic” were searched in PubMed, Embase, Web of Science, and the Cochrane Library. The retrieval time is of the establishment in the database in February 2021, and all the literature on the application of systematic nursing in endoscopic removal of colorectal polyps in children is collected. Take PubMed as an example, the retrieval strategy is shown in Table 1.

**Table 1:** Search strategy in PubMed database.

Number	Search terms
#1	Systematic nursing [Title/Abstract]
#2	Comprehensive nursing [Title/Abstract]
#3	nursing intervention [Title/Abstract]
#4	#1 OR #2 OR #3
#5	Colonic polyps [MeSH]
#6	Polyp, Colonic [Title/Abstract]
#7	Colorectal polyp [Title/Abstract]
#8	Pediatric polyp [Title/Abstract]
#9	#5 OR #6 OR #7 OR #8
#10	Endoscopy [Title/Abstract]
#11	Endoscopic [Title/Abstract]
#12	#10 OR #11
#13	#4 AND #9 AND #12

### 2.7 Data screening and extraction

The data will be extracted independently by the 2 researchers, the information will be recorded in the data extraction form, and their differences will be resolved with the help of a third reviewer. The detailed information is extracted as follows:

(1) Clinical study (title, first author, publication date, sample size, sex ratio, average age, length of stay, type of operation);
(2) intervention measures (type of care, nursing plan, duration, follow-up time) between treatment group and control group;
(3) risk bias assessment factors in randomized controlled trials;
(4) outcome indicators.

**Figure 1:**
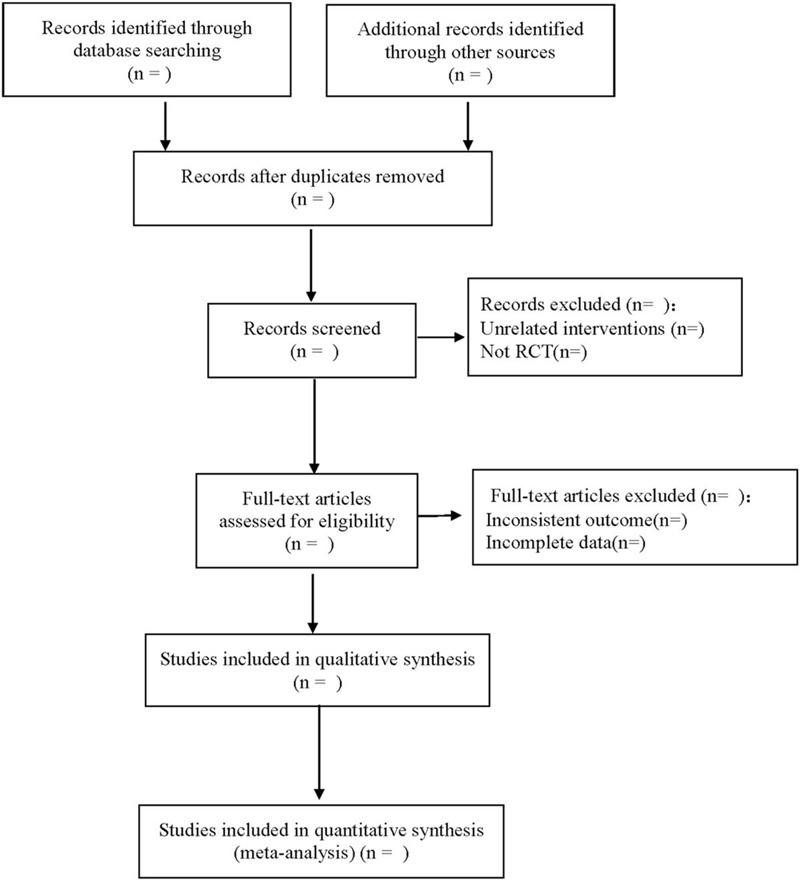
Flow diagram.

The literature screening process is shown in Figure 1.

### 2.8 Literature quality assessment

The quality evaluation of the literature is completed independently by 2 researchers according to the Cochrane5.1.0 system evaluation manual. When there is disagreement, the third researcher participates in the discussion, and finally decides the overall quality of the literature. Evaluation indicators include random sequence generation, distribution hiding, blind method, integrity of result data, selective reporting of research results, and other sources of bias. According to these indicators, the included literature was evaluated as “high risk of bias,” “low risk of bias,” and “unknown.”

### 2.9 Statistical analysis

#### 2.9.1 Data analysis and processing

RevMan 5. 3 software is used for meta-analysis. The heterogeneity of each literature is judged by test and *I*^2^, and the heterogeneity of the effect value is analyzed. If *P* *>* .1*, I*^2^ *<* 50%, it shows that the heterogeneity among the studies is low, so a fixed model is used for analysis; if *P* *<* .1*, I*^2^ *≥* 50%, there is obvious heterogeneity among the studies. The sources of heterogeneity are analyzed and the random effect model is used for analysis. The measurement data are expressed as the weighted mean difference or the standard mean difference and 95% confidence interval (CI), and the counting data are expressed as relative ratio and 95% CI.

#### 2.9.2 Dealing with missing data

If the data of the required study is incomplete or not reported in the study, the researcher will contact the first author or other author of the study by phone or email. If the required data are not available, we will use descriptive analysis instead of meta-analysis and exclude these studies if necessary.

#### 2.9.3 Subgroup analysis

We will further explore the impact of systematic nursing on children according to the type of operation, nursing plan, patient age, and other factors.

#### 2.9.4 Sensitivity analysis

According to the recommendations of the Cochrane manual, we will conduct a sensitivity analysis of each outcome index. In order to test the stability of meta-analysis results of indicators, an one-by-one elimination method will be adopted for sensitivity analysis.

#### 2.9.5 Assessment of reporting biases

If there are more than 10 studies, funnel chart is used to evaluate whether there is publication bias. Moreover, Egger and Begg test are used for the evaluation of potential publication bias.

#### 2.9.6 Evidence quality evaluation

We will use the Grading of Recommendation Assessment, Development, and Evaluation scoring method to grade the evidence of the outcome index.^[18]^ The evaluation content includes bias risk, indirectness, inconsistency, inaccuracy and publication bias, and the quality of evidence will be rated as high, medium, low, or very low.

## 3 Discussion

Because the mental development of pediatric patients is not mature, their treatment and clinical nursing are difficult, so they need to pay more attention to it. Clinical nursing of colorectal polyps in children should be fully considered before, during, and after operation.^[19]^ Due to the lack of understanding of the disease before operation, children and their families may produce various factors that affect emotion and affect treatment and nursing, so appropriate psychological nursing should be given to children before operation and adequate preparation should be done before operation; the focus of intraoperative nursing is to assist doctors to carry out a series of surgical cooperation. Postoperative nursing should not only carry out psychological nursing for children's discomfort, but also carefully observe the changes of vital signs, prevent and cure complications, and control their diet, exercise, and living habits.^[20]^

The implementation of practical nursing intervention can reduce the psychological stress reaction of patients, help patients and their families build confidence, improve their ability to cope with and solve problems, and make children go through the operation period smoothly, improve the success rate of polypectomy under electronic colonoscopy. Previous studies have confirmed that systematic nursing has obvious significance in effectively reducing the negative reaction of children, improving the degree of cooperation of children's families, shortening the time of operation, and improving the curative effect of operation.^[21]^ Perioperative standardized nursing plays a decisive role in whether patients can effectively achieve a positive impact. Through this systematic evaluation, we will provide reliable evidence-based basis for systematic nursing care of children with colorectal polyps undergoing endoscopic resection.

However, this systematic review has some limitations. Due to the age, polyps type, specific content of care and other factors will increase the possibility of heterogeneity; due to the limitations of language retrieval, we will only include Chinese and English literature, and may ignore the research in other languages and regions.

## Author contributions

**Data collection:** Zhenyin Dong and Guizhen Li and Meigui Wang.

**Resources:** Guizhen Li and Meigui Wang.

**Software operating:** Tingting Wang and Chengpeng Jiang.

**Supervision:** Meigui Wang and Tingting Wang.

**Funding support:** Chengpeng Jiang.

**Writing – original draft:** Zhenyin Dong and Guizhen Li and Meigui Wang.

**Writing – review & editing:** Zhenyin Dong and Chengpeng Jiang.
